# The feasibility of emotion-focused therapy for binge-eating disorder: a pilot randomised wait-list control trial

**DOI:** 10.1186/s40337-020-00358-5

**Published:** 2021-01-06

**Authors:** Kevin Glisenti, Esben Strodl, Robert King, Leslie Greenberg

**Affiliations:** 1 School of Psychology and Counselling, Queensland University of Technology, Faculty of Health, Brisbane, Queensland Australia; 2 Department of Psychology, York University, Faculty of Health, Toronto, Canada

**Keywords:** Emotion-focused therapy, Binge-eating disorder, Feasibility, Emotion regulation, Pilot randomised control trial

## Abstract

**Background:**

Research into psychotherapy for binge-eating disorder (BED) has focused mainly on cognitive behavioural therapies, but efficacy, failure to abstain, and dropout rates continue to be problematic. The experience of negative emotions is among the most accurate predictors for the occurrence of binge eating episodes in BED, suggesting benefits to exploring psychological treatments with a more specific focus on the role of emotion. The present study aimed to explore the feasibility of individual emotion-focused therapy (EFT) as a treatment for BED by examining the outcomes of a pilot randomised wait-list controlled trial.

**Methods:**

Twenty-one participants were assessed using a variety of feasibility measures relating to recruitment, credibility and expectancy, therapy retention, objective binge episodes and days, and binge eating psychopathology outcomes. The treatment consisted of 12 weekly one-hour sessions of EFT for maladaptive emotions over 3 months. A mixed model approach was utilised with one between effect (group) using a one-way analysis of variance (ANOVA) to test the hypothesis that participants immediately receiving the EFT treatment would demonstrate a greater degree of improvement on outcomes relating to objective binge episodes and days, and binge eating psychopathology, compared to participants on the EFT wait-list; and one within effect (time) using a repeated-measures ANOVA to test the hypothesis that participation in the EFT intervention would result in significant improvements in outcome measures from pre to post-therapy and then maintained at follow-up.

**Results:**

Recruitment, credibility and expectancy, therapy retention outcomes indicated EFT is a feasible treatment for BED. Further, participants receiving EFT demonstrated a greater degree of improvement in objective binge episodes and days, and binge eating psychopathology compared to EFT wait-list control group participants. When participants in the EFT wait-list control group then received treatment and outcomes data were combined with participants who initially received the treatment, EFT demonstrated significant improvement in objective binge episodes and days, and binge eating psychopathology for the entire sample.

**Conclusions:**

These findings provide further preliminary evidence for the feasibility of individual EFT for BED and support more extensive randomised control trials to assess efficacy.

**Trial registration:**

The study was retrospectively registered with the Australian New Zealand Clinical Trials Registry (ACTRN12620000563965) on 14 May 2020.

## Plain English summary

Research into psychotherapy for binge-eating disorder (BED) has focused mainly on cognitive-behavioural therapies, but efficacy, failure to abstain, and dropout rates continue to be problematic. The experience of negative emotions is among the most accurate predictors for the occurrence of binge eating episodes in BED, suggesting benefits to exploring psychological treatments with a more specific focus on the role of emotion. This pilot randomised wait-list control trial aimed to investigate the feasibility of emotion-focused therapy (EFT) for BED using a variety of measures relating to recruitment, credibility and expectancy, therapy retention, objective binge episodes and days, and binge eating psychopathology outcomes.. Initially, participants were randomly allocated to an immediate EFT treatment or EFT wait-list control group. The treatment consisted of 12 weekly one-hour sessions of EFT over 3 months with 21 participants. Recruitment, credibility and expectancy, and therapy retention outcomes indicated EFT is a feasible treatment for BED. Further, participants immediately receiving the EFT demonstrated a greater degree of improvement in binge episodes, the number of days on which binge episodes occurred, and binge eating symptoms compared to participants in EFT the wait-list control group. Participants in the EFT wait-list control group then received treatment and outcomes data were combined with participants who initially received the treatment. EFT resulted in significant improvements in binge episodes and the number of days on which binge episodes occur, and binge eating psychopathology for the entire sample. These findings provide further preliminary evidence for the feasibility of individual EFT for BED, and support for more extensive randomised control trials to assess efficacy.

Binge-eating disorder (BED) is the most prevalent of all the eating disorders [1]. There is an estimated prevalence rate of 2.5 to 4.5% in females and 1.0 to 3.0% in males based on international data [2] and a 3-month prevalence rate of 5.58% based on Australian data [3]. The core symptoms include recurrent episodes of binge eating while experiencing a sense of lack of control in the absence of compensatory strategies [4]. Not surprisingly, many individuals with BED also have comorbid emotional disorders [5] including anxiety [6, 7] and depression [8, 9]. For BED, both The National Institute of Clinical Excellence (NICE) in the United Kingdom and the American Psychiatric Association guidelines suggest that cognitive behaviour therapy (CBT) is the psychological treatment of choice, with interpersonal therapy (IPT) and dialectical behaviour therapy (DBT) serving as second-line interventions [10, 11].

In a recent meta-analysis, Hilbert et al. [12] explored the efficacy of psychological treatments for BED. Psychological therapy, mostly CBT, demonstrated large effect sizes for the reduction of binge episodes and abstinence from binge eating in RCTs with inactive control groups, followed by structured self-help with medium-to-large effects when compared with wait-list. There was limited evidence for the of one treatment approach over another in RCTs with active control groups, and more extensive research with a focus on longer-term maintenance of therapy gains, efficacy, mechanisms of change and complex models of care was recommended [12]. In another more recent meta-analysis, Linardon [13] estimated the prevalence of patients with BED who achieved binge-eating abstinence following psychological or behavioural treatments. The most common treatment delivered was CBT (either in a clinician-led or guided self-help format), and other interventions include behavioural weight loss, behavioural weight loss combined with CBT, IPT, DBT, behaviour therapy, non-specific supportive therapy, mindfulness, psychodynamic therapy, and a combined psychotherapy approach. The total weighted percentage of treatment-completers who achieved abstinence at posttreatment was 50.9 and 50.30% at follow-up. The highest abstinence rate was observed in IPT, and clinician-led group treatments produced significantly higher posttreatment (but not follow-up) abstinence estimates than guided self-help treatments. The meta-analysis demonstrated that 50% of patients with BED do not fully respond to treatment, and there is, therefore, a need to explore other psychotherapies to improve outcomes [13].

Individuals with BED often experience difficulties with deficits in emotion regulation which can be defined as the “… attempt to influence which emotions we have, when we have them, and how these emotions are experienced or expressed.” ( [14], p. 224). Several emotion regulation theories have been proposed to explain eating-related problems. For example, emotional eating theory conceptualises eating as a coping strategy in response to emotional distress [15, 16]; escape theory presumes a reduction of negative affect while bingeing [17]; affect regulation theory [18] assumes an improvement in negative affect after bingeing; and emotional arousal theory conceptualises overeating as being evoked by emotional arousal in order to reduce the level of arousal [19]. Given each of these theories includes negative emotions as a trigger for binge eating (i.e., trigger component) and/or down-regulation of negative emotions (i.e., relief component) while or after binge eating, [20] proposes an ‘emotion regulation model’ of binge eating which incorporates both components and includes the whole emotion regulation process. Binge eating occurs in response to intolerable emotional experiences in the absence of more adaptive coping mechanisms [21] and represents an effort by an individual to regulate emotion by numbing, avoiding or soothing negative or overwhelming affect [22]. It occurs in the absence of effective regulation skills related to experiencing and differentiating as well as attenuating and modulating emotions [23], and individuals with BED experience more intense emotions and more significant difficulties in emotion regulation than individuals without BED [24].

Given that the experience of negative emotions is amongst the best predictors for the occurrence of binge eating episodes in BED [25], outcomes could be improved by psychological treatments with a more specific focus on the role of emotion. Indeed, innovative treatments for BED with a more specific focus on emotion are emerging, including integrative cognitive-affective therapy [26] and emotion-focused cognitive-behavioural therapy [27]. Emotion-focused therapy (EFT) is a compelling treatment for eating disorders and offers a unique framework for understanding the pathogenesis of emotional difficulties (either under-regulating or over-regulating affect) in this population [28]. The goal of EFT is to assist clients in 1) identifying and accepting primary emotions (their very first emotional response to a stimulus situation) from secondary emotions (a response that obscures their primary response) 2) processing primary maladaptive negative emotions by attending to and increasing awareness and expression of these primary maladaptive emotions; learning to tolerate and regulate painful underlying experience; reflecting upon and make meaning of emotion by symbolising emotional experience in words; and transforming maladaptive emotions by activating healthy, adaptive emotions together with their associated needs and action tendencies [29].

According to the EFT model, emotion organises experience through emotion schemes, which are constructed from lived emotional experience [30]. Central mechanisms of change in EFT include 1) identifying primary maladaptive emotion that are obscured by secondary symptomatic emotions and having arrived at these emotions, the use of adaptive emotion to transform maladaptive emotion schemes that are understood to generate chronic enduring pain and maintain secondary symptomatic behaviour and rigid and maladaptive modes of responding to experience [31], and a successful therapeutic relationship, in which the client feels empathically heard, understood, supported and safe [32]. The feasibility and efficacy of EFT have been established in various disorders including depression (e.g., [33–35]); complex trauma (e.g., [36–38]); and are emerging for anxiety [39–41]. While there is a growing body of literature exploring the use of EFT for eating disorders (e.g., [28, 29, 42–45]), this literature has generally included mixed samples of BED, Anorexia Nervosa (AN) and BN with limited research focusing specifically on BED.

To date, only one study has examined the feasibility and efficacy of EFT specifically for BED; however, this was group therapy based. In a non-randomised observational study, Compare and Tasca [46] compared the outcome of 20 weeks of emotionally focused group therapy (EFGT), aimed at helping clients change how they experienced and used their emotions, with combined therapy (CT) of EFGT plus dietary counselling, which sought to lower energy-dense food intake in 118 obese adults with BED. Participants were assigned to EFGT or CT based on consensus among clinicians. Binge episodes and weight significantly declined during both treatments; however, compared to EFGT, CT resulted in more rapid weight loss across the weeks of therapy. The dropout rate for EFGT was only 6%. Further, to date, only one study to date has examined the feasibility of individual therapy based EFT for BED [47]. This study involved the use of a multiple baseline case series design in which individual EFT over 12 weeks, was applied to six female adult participants with BED, with follow-ups at 2, 4- and 8-weeks posttreatment. All cases experienced reliable recovery from binge-eating psychopathology and also a significant decrease in binge-eating frequency. There was reliable improvement or recovery for eating and shape concerns for all cases, and improvement on weight concern for the majority of cases; and all cases experienced reliable recovery or improvement in overall emotion regulation. Most cases that were in the clinical range for anxiety at pre-treatment recovered and all cases experienced reliable improvement in, or recovery from, depression. Three of the six cases experienced reliable recovery or improvement in alexithymia. There were no treatment dropouts.

While there is emerging preliminary evidence for the use of EFT for BED, a pilot trial to further test feasibility is required. Arain, Campbell, Cooper and Lancaster [48] provide a useful model upon which the feasibility of a pilot study can be assessed. This model suggests that feasibility studies incorporate research conducted before the main study in order to identify important parameters needed to design the main study. These can include participant willingness to be randomised, the number of eligible participants, and other aspects such as therapy retention rates. Further, it has been proposed that the purpose of a pilot study is to examine the feasibility of an approach intended to be used in a larger-scale study, through evaluating recruitment, randomisation and retention, in addition to assessment procedures, new methods, and implementation of novel interventions [49]. The current study presents results from a pilot randomised wait-list control trial to assess the feasibility of individual EFT for BED. It was hypothesised that:

1. Participants would be willing to be randomised, and an appropriate number of participants would be deemed eligible for the research.
2. Participation in EFT would result in higher participant treatment credibility and expectancy for improvement scores.
3. There would be a lower dropout rate using EFT for BED compared to more commonly used psychological treatment approaches.
4. Immediate EFT treatment group participants would demonstrate a significantly greater degree of improvement in objective binge episodes, the number of days on which objective binge episodes occurred, and binge eating psychopathology, compared to participants in an EFT wait-list control group who had not received the treatment.
5. The total sample (i.e. the immediate EFT treatment group who initially received treatment, and also EFT wait-list group post receiving treatment), would experience significant improvements in objective binge episodes, the number of days on which objective binge episodes occurred, and binge eating psychopathology.

## Method

### Design

This study is a pilot randomised wait-list control trial designed to explore the feasibility of individual emotion-focused therapy (EFT) as a treatment for BED. It builds upon findings from an initial multiple baseline case series design of EFT for BED [47]. The sample size for the current study was calculated using the outcome measure of changes in binge eating episodes from the initial case series as a basis. This showed an effect size of Cohen’s d = 2.91. Using GPower with this effect size, alpha at .05, power at .95, using repeated measures ANOVA between groups design with 3 time points, yielded a required total sample size of just 6 participants. Binge eating disorder treatment pilot study sample sizes vary, e.g., 7 [27], 10 [50], 36 [51], and 41 [52]. We chose a sample size clearly larger than what was required by our power analysis from the initial case series, and at the mid-point of the aforementioned pilot studies.

**Fig. 1:**
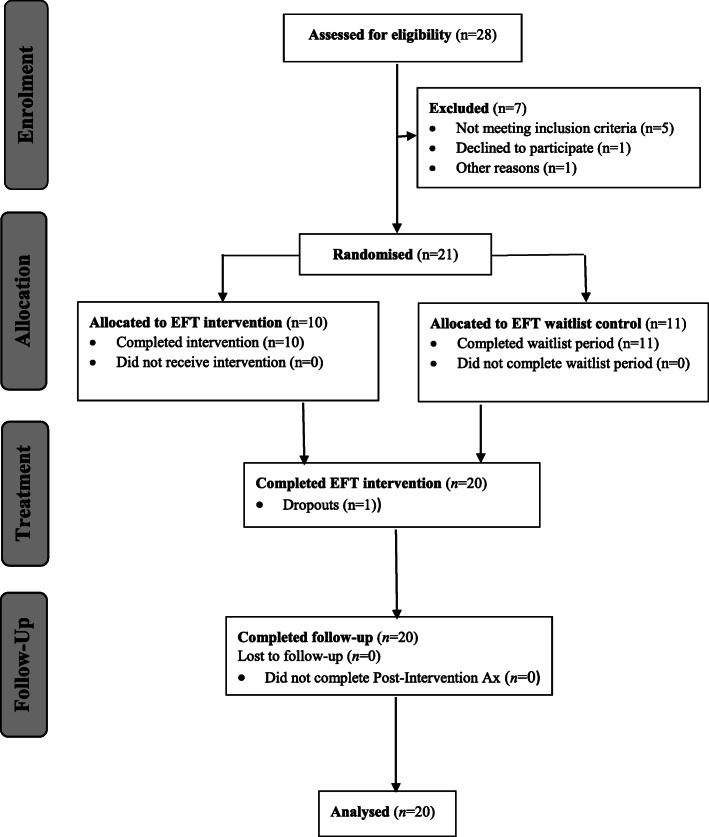
CONSORT flow diagram

Participants were initially randomly allocated to either an immediate EFT treatment group (12 weekly EFT sessions) or an EFT wait-list control group (12-week clinical monitoring preceding 12 weekly EFT sessions) using a block randomisation method [53]. This is a commonly used technique in clinical trial design which reduces bias and achieves sample size balance when allocating participants to treatment groups. It is particularly useful for smaller sample sizes and increases the probability that each allocation arm will contain an equal number of individuals by sequencing participant assignments by block. This project was approved by the Queensland University of Technology (QUT) University Human Research Ethics Committee (UHREC) and met the requirements of the National Statement on Ethical Conduct in Human Research (2007). The UHREC Reference number is 1700000986, and all participants provided written informed consent. Consolidated Standards of Reporting Trials (CONSORT) guidelines were fully adhered to – See Fig. 1.

### Participants

Participants were recruited from local General Practitioners/Primary Care Physicians. Inclusion criteria included the following: being between 18 and 65 years of age, meeting the Diagnostic and Statistical Manual of Mental Disorders: DSM-V American Psychiatric Association – DSM-5 [4] diagnostic criteria for BED, and possessing sufficient English language skills to provide informed consent and participate in the study without translation. The exclusion criteria included current psychosis, intellectual disability, high suicide risk, drug or alcohol abuse, concurrent treatment for obesity, pregnancy, and the presence of AN or BN. The total sample consisted of 21 participants, of whom 17 were female, and 4 were male. The average age was 44.52 (*SD* = 11.89) years and the average age at first binge 18.23 (*SD* = 8.07) years. Ten participants were married or living with someone as married, 5 separated, 3 never married, 2 divorced and 1 widowed. Six participants had graduated four-year college, 5 graduated two-year college or trade school, 4 completed grades 7–12 (without graduating high school), 3 graduated high school or high school equivalent, 2 partially completed college/trade school and 1 postgraduate/professional school. Eleven participants were employed full-time, 5 part-time employment, 2 keeping house or caregiving full time, 2 in school/training and 1 disabled.

### Measures

#### Pretherapy assessment measures

Pretherapy diagnostic assessment of BED was based on the Structured Clinical Interview for DSM-5-Research Version - SCID-5-RV [54]. At present, there is limited reliability or validity data available for the SCID-5-RV; however, it has demonstrated internal consistency (.80) and test-retest reliability [55]. Previous versions of Structured Clinical Interview for DSM-IV Axis I Disorders - SCID-I [56], however, have demonstrated a high level of inter-rater reliability (k = .75) for symptoms and 90% accuracy in diagnosis [57].

### Feasibility measures

#### Recruitment

Data were obtained in relation to various aspects of recruitment, including participant willingness to be randomised, and the number of eligible participants during the recruitment process.

#### Credibility and expectancy

Treatment credibility and participant expectancy for improvement were measured using the Credibility and Expectancy Questionnaire – CEQ [58]. The CEQ is a 6-item self-report measure of treatment expectancy and rationale credibility. Items 1 to 4 are rated based on cognitive appraisal, i.e., asking participants what they think will happen (e.g., “At this point, how logical does the treatment offered to you seem?” and “At this point, how useful do you think the treatment will be in reducing your binge eating disorder symptoms?”), and items 5–6 are rated based on affective aspects of beliefs, i.e., asking participants what they feel will happen (e.g., “At this point, how much do you really feel that therapy will help you reduce your binge eating disorder symptoms?” and “By the end of the therapy period, how much improvement in your binge eating disorder symptoms do you really feel will occur?”). The original CEQ contains four questions on credibility using a 9-point Likert scale, where 1 = not at all and 9 = very much or very useful, and two questions on expectancy using a scale from 0 to 100% in 10% increments. Given the use of two scales during administration, a composite score is derived for each factor by initially standardising the individual items and then summing those items for each factor. The CEQ has demonstrated adequate test-retest reliability of the expectancy (.82) and credibility factor (.75). The combined overall rating of the treatment rationale resulted in a composite index high test-retest reliability of (.83) and adequate internal consistency [α = .85] [58].

#### Therapy retention

Participants who completely discontinued attendance were considered dropouts.

##### Objective binge episodes and days

Changes in objective binge episodes and days (occurrence over the previous 7 days) were assessed using items from the Eating Disorder Examination Questionnaire – EDE-Q-6.0 [59]. The EDE-Q-6.0 is a self-report measure of eating disorder psychopathology based on the Eating Disorder Examination Interview [60]. It is a widely used measure of eating disorder attitudes and behaviours in both community and clinical populations [61]. The EDE-Q-6.0 also provides frequency data on the number of episodes of the eating disorder behaviours and the number of days on which the behaviour occurred. The items used to measure objective binge episodes (i.e., a discrete episode of overeating of an objectively large amount of food associated with a feeling of loss of control) in the current study were: “Over the past 7 days how many times have you eaten what other people would regard as an unusually large amount of food (given the circumstances)?” and “On how many of these times did you have a sense of having lost control over your eating (at the time that you were eating)?”. The item used to measure the number of days objective binge episodes occurred was “Over the past 7 days, on how many days have such episodes of overeating occurred (i.e., you have eaten an unusually large amount of food and have had a sense of loss of control at the time)?”. The EDE-Q-6.0 has received support as a reliable and valid measure of eating-related pathology and specific disordered eating behaviours [62, 63]. Test-retest reliability across studies ranges from 0.66 to 0.94 for scores on the four subscales [64]. The EDEQ-Q-6.0 has demonstrated acceptable levels of internal consistency (α = .90) for the total score in a clinical sample [65]. There are no standardised clinical cut-offs [59].

##### Binge eating psychopathology

Changes in binge-eating psychopathology were assessed using the Binge Eating Scale – BES [66]. The BES is a commonly used self-report screening tool for binge eating in clinical practice and research. A total of 16 items are rated using 3–4 separate responses assigned a numerical value. An example of an item is “(a.) I feel capable to control my eating urges when I want to; (b.) I feel like I have failed to control my eating more than the average person; (c.) I feel utterly helpless when it comes to feeling in control of my eating urges; (d.) Because I feel so helpless about controlling my eating I have become very desperate about trying to get in control”. Total scores range from 0 to 46, with higher scores indicating more severe binge-eating symptoms. The BES has demonstrated high test-retest reliability (*r* = .71) and internal consistency (α = .85) in an obese population [67], good 2-week test-retest reliability (*r* = .87) in a behavioural weight loss sample [68], high internal consistency (*α* = .91) in a BED sample [69], and good construct reliability and convergent validity [70]. Standardised cut-off scores are as follows: ≤ 17 = no binge eating, 18–26 = mild to moderate binge eating, and ≥ 27 = severe binge eating [66].

#### Procedures

Participants were initially telephone-screened for BED based on diagnostic criteria, according to DSM-5 [4]. Twenty-eight participants were telephone-screened, of which five did not meet the diagnostic criteria for BED. Twenty-three participants meeting the diagnostic criteria for BED then completed the SCID-5-RV administered by the same research assistant with training in clinical psychology. All met the inclusion criteria, but 1 participant chose not to participate due to being unable to commit fully to weekly treatment sessions, and 1 participant did not respond to contact attempts. Twenty-one participants were randomly allocated to either an immediate EFT intervention or 12-week EFT wait-list using a block randomisation method, by a statistician independent to the research team.

Participants allocated to the immediate EFT intervention completed the BES and EDE-Q-6.0 pretherapy (Week 0). The BES and EDE-Q-6.0 were completed weekly (Weeks 1–12), and CEQ at weeks 1, 3, 5, 7, 9 and 11 during treatment. The BES and EDE-Q-6.0 were completed at follow-up (Weeks 16, 20 and 24). Participants allocated to the EFT wait-list control completed the BES and EDE-Q-6.0at pretherapy (Week 0), and then again 12 weeks later post wait-list period completion. Wait-list control participants then commenced 12 weekly treatment sessions and followed the same protocol as participants initially allocated to the immediate EFT treatment intervention. The therapist was blind to all assessments and randomisation of the participants.

#### Treatment

Treatment incorporated 12 weekly one-hour sessions of EFT for maladaptive emotions over 3 months. The treatment manual was initially adapted from [45] by [47] in a series of case studies exploring the use of individual EFT to treat BED. Phase 1 of the treatment focused on promoting awareness of emotions, welcoming and accepting emotions, putting emotions into words, and identifying primary emotions. Phase 2 focused on evaluating whether the primary emotion was adaptive or maladaptive, identifying destructive emotions, accessing other adaptive emotions and needs, and transforming maladaptive emotion schemes. Six main marker guided interventions were used in treatment in line with EFT protocol (30, 323). These were: 1. Empathic attunement and validation for vulnerability and establishing the therapeutic alliance 2. Evocative unfolding for problematic reactions 3. Experiential focusing for unclear feelings 4. Two-chair work for self-critical splits 5. Two-chair work for self-interruptive splits and 6. Empty chair work for unfinished business.

### Therapist

The therapist was the first author, a Clinical Psychologist with 25 years of practice experience who had undergone Level 1, 2 and 3 training in EFT at the York University Psychology Clinic with the primary developer of this approach, Distinguished ProfessorEmeritus, Leslie Greenberg. The therapist had approximately 4 years of EFT-specific practice experience before the study and was not involved in the initial treatment/wait-list randomisation process, data collection before or during the study, or data analysis until after the study. Supervision was provided by Distinguished Professor Emeritus, Leslie Greenberg, who was also a co-author of the original treatment manual used as a basis for therapy within the current study. Adherence to EFT protocol was reviewed - and rectified where necessary - during supervision based on video recordings of study treatment sessions.

### Statistical analyses plan

Initially, a one-way ANOVA was conducted in relation to any significant demographic differences between participants randomly allocated to the immediate EFT and EFT wait-list control group at baseline. Following this, a mixed model approach was utilised with one between effect (group) using a one- way ANOVA to test the hypothesis that participants immediately receiving the EFT treatment would demonstrate a greater degree of improvement on outcome measures relating to objective binge episodes and days, and binge eating psychopathology compared to participants on the EFT wait-list; and one within effect (time) using a repeated-measures ANOVA to test the hypothesis that participation in the EFT intervention would result in significant improvements in outcome measures from pre to post-therapy and then maintained at each follow-up period, for the total sample. Missing data were managed using pairwise deletion.

## Results

### Demographics

Table 1 outlines the demographics of participants who completed treatment at baseline.

**Table 1:** Participant demographics at baseline for treatment completers (*n* = 20)

Case	Allocation	Gender	Age	Marital Status	Education Status	Employment Status	Age First Binge	Objective Binge Episodes^a.^	Binge and Loss Control Days^a.^
1	Treatment	Female	36	Separated	2-year college/trade school	School/training	33	7	7
2	Treatment	Female	57	Separated	4-year college	Part-time job	19	4	4
3	Treatment	Female	28	Never married	Part college/trade school	School/training	14	20	6
4	Treatment	Female	23	Married or with someone	High school or equivalent	Keeping house	10	5	5
5	Treatment	Male	58	Separated	High school or equivalent	Disabled	15	1	1
6	Treatment	Female	38	Widowed	4-year college	Keeping house	10	7	5
7	Treatment	Female	32	Married or with someone	4-year college	Full-time job	15	6	6
8	Treatment	Female	45	Divorced or annulled	Part graduate school	Full-time job	24	16	5
9	Treatment	Female	61	Married or with someone	Grades 7–12	Full-time job	8	2	2
10	Treatment	Male	42	Married or with someone	2-year college/ trade school	Full-time job	13	1	1
11	Waitlist	Male	47	Divorced or annulled	4-year college	Full-time job	18	9	7
12	Waitlist	Female	65	Married or with someone	2-year college/trade school	Part-time job	28	5	5
13	Waitlist	Female	42	Married or with someone	4-year college	Full-time job	16	10	7
14	Waitlist	Female	36	Married or with someone	Grades 7–12	Full-time job	15	3	2
15	Waitlist	Female	32	Never married	Part college/trade school	Full-time job	13	2	2
16	Waitlist	Female	50	Married or with someone	Grades 7–12	Part-time job	28	3	2
17	Waitlist	Male	62	Married or with someone	2-year college/trade school	Part-time job	15	5	5
18	Waitlist	Female	45	Never married	2-year college/ trade school	Part-time job	18	1	1
19	Waitlist	Female	41	Separated	High school or equivalent	Full-time job	18	5	6
20	Waitlist	Female	38	Separated	4-year college	Full-time job	13	8	7

^a^In previous 7 days

### Assessment of feasibility

#### Recruitment

Participants were recruited over a period of 9 months, and this phase was protracted due to a lower than expected take-up rate. Reports from general practitioners/primary care physicians indicated the one main reason for this was the relatively lower percentage of patients with binge eating disorder within the medical practices. Twenty-eight participants were assessed for eligibility, and 5 five did not meet the diagnostic criteria for BED. All remaining participants met the inclusion criteria, but 1 chose not to participate due to being unable to commit fully to weekly treatment sessions, and 1 did not respond to contact attempts. Twenty-one participants were randomly allocated to either an immediate EFT intervention or 12-week EFT wait-list using a block randomisation method. While 3 participants allocated to an EFT wait-list expressed disappointment about not proceeding to treatment immediately, each expressed a strong willingness to continue involvement in the study.

#### Credibility and expectancy

The CEQ demonstrated adequate internal consistency (α = .86). Mean CEQ credibility scores remained high during early, mid and late therapy: 7.40 (SD = 1.30) at Week 1, 8.01 (SD = .58) at Week 7 and 7.85 (SD = 1.16) at Week 11. Mean CEQ expectancy scores also remained high during early, mid and late therapy: 6.86 (SD = 1.16) at Week 1, 7.13 (SD = 1.30) at Week 7 and 7.06 (SD = 1.32) at Week 11 (See Table 2.) Further, significant differences were not identified in mean treatment credibility and expectancy scores from early to mid-therapy, mid to late therapy or early to late therapy.

**Table 2:** CEQ early, mid and late therapy mean scores for the entire sample (*n* = 20)

	Early therapy^a.^	Mid Therapy^b.^	Late Therapy^c.^	Early therapy to Mid therapy		Mid therapy to Late therapy		Early therapy to Late therapy	
	*M (SD)*	*M (SD)*	*M (SD)*	*t-Test*	*D*	*t-Test*	*d*	*t-Test*	*d*
CEQ credibility	7.40 (1.30)	8.01 (.58)	7.85 (1.16)	−2.04	.45	.93	−.16	−1.53	.32
CEQ expectancy	6.86 (1.16)	7.13 (1.30)	7.06 (1.32)	−1.03	.23	.29	−.07	−.75	.17

*CEQ* Credibility Expectancy Questionnaire; Week 1^a.^ Week 7^b.^ Week 11^c^

#### Therapy retention

One participant (4.76%) dropped out after Week 4 of the EFT treatment for family health reasons. All completing participants attended all sessions.

#### Immediate EFT intervention group versus EFT wait-list control group

##### Demographics

No significant demographic differences were found between the immediate EFT intervention and EFT wait-list control groups in relation to mean age (years), mean age at first binge (years), gender, marital status, education, and employment status. See Table 3.

**Table 3:** Participant demographics by treatment group at randomization for treatment completers (*n* = 20)

	Immediate EFT Group (*n* = 10)	EFT Waitlist Group (*n* = 10)		
Demographic	*M* (SD) or *f*	*M* (SD) or *f*	*t* (df) or Χ^2^	*p*-value
Age (years)	42.00 (13.16)	45.80 (10.73)	.67 (18)	.49
Age first binge (years)	16.10 (7.50)	18.20 (5.49)	.73 (18)	.49
Gender				
Male	2	2	.000	1.00
Female	8	8		
Marital Status				
Married or with someone	4	5	1.64	.80
Widowed	1	0		
Divorced or annulled	1	1		
Separated	3	2		
Never married	1	2		
Education				
Grades 7–12	1	2	1.86	.87
High school or equivalent	2	1		
Part college/trade school	1	1		
2-year college/trade school	2	3		
4-year college	3	3		
Part graduate school	1	0		
Employment Status				
Full-time job	4	6	7.20	.13
Part-time job	1	4		
Keeping house	2	0		
School/training	2	0		
Disabled	1	0		

##### Objective binge episodes

Significant differences were found between the immediate EFT intervention group mean scores from Week 0 to Week 12 of treatment, and EFT wait-list control group mean scores from Week 0 to Week 12 of the wait-list period, in relation to objective binge episodes. Compared with the EFT wait-list control group, the immediate EFT intervention group showed significantly greater reductions in objective binge episodes with a large treatment effect (*d* = .98). See Table 4.

**Table 4:** Mean Immediate EFT Group (*n* = 10) and EFT Waitlist Group (*n* = 10) EDEQ and BES pretherapy and posttherapy/postwaitlist scores

	Immediate EFT Group	EFT Waitlist Group	Immediate EFT Group	EFT Waitlist Group				
	Pretherapy^a^	Pretherapy	Post Therapy^b.^	Post Waitlist^c^				
	*M* (*SD*)	*M* (*SD*)	*M* (*SD*)	*M* (*SD*)	*F*	*p*-value	η_p_^2^	Cohen’s *d*
Outcome								
EDEQ objective binge episodes	6.90 (6.33)	5.10 (3.03)	2.90 (2.88)	5.10 (2.84)	6.85	.017	.276	.98
EDEQ objective binge episode days	4.20 (2.15)	4.40 (2.41)	1.50 (1.64)	4.70 (2.31)	40.09	.001	.690	1.39
BES binge eating psychopathology	25.60 (9.65)	28.30 (6.93)	20.70 (11.77)	29.20 (7.98)	12.12	.003	.402	.62

*EDEQ* Eating Disorders Examination Questionnaire *BES* Binge Eating Scale; Week 0^a.^ Week 12 of treatment^b.^ Week 12 of wait list^c^

##### Objective binge days

Significant differences were also found between the immediate EFT intervention group mean scores from Week 0 to Week 12 of treatment, and EFT wait-list control group mean scores from Week 0 to Week 12 of the wait-list period, in relation to objective binge episodes days. When compared with the EFT wait-list control group, the immediate EFT intervention group experienced significantly greater reductions in objective binge episode days with a very large treatment effect (*d* = 1.39). See Table 4.

##### Binge eating psychopathology

Significant differences were also found between the immediate EFT intervention group mean scores from Week 0 to Week 12 of treatment, and EFT wait-list control group mean scores from Week 0 to Week 12 of the wait-list period, in relation to binge eating psychopathology. Compared with the EFT wait-list control group, the immediate EFT intervention group displayed significantly greater reductions in binge eating psychopathology with a moderate treatment effect (d = .62).

### Combined immediate EFT intervention group and EFT wait-list control group

**Fig. 2:**
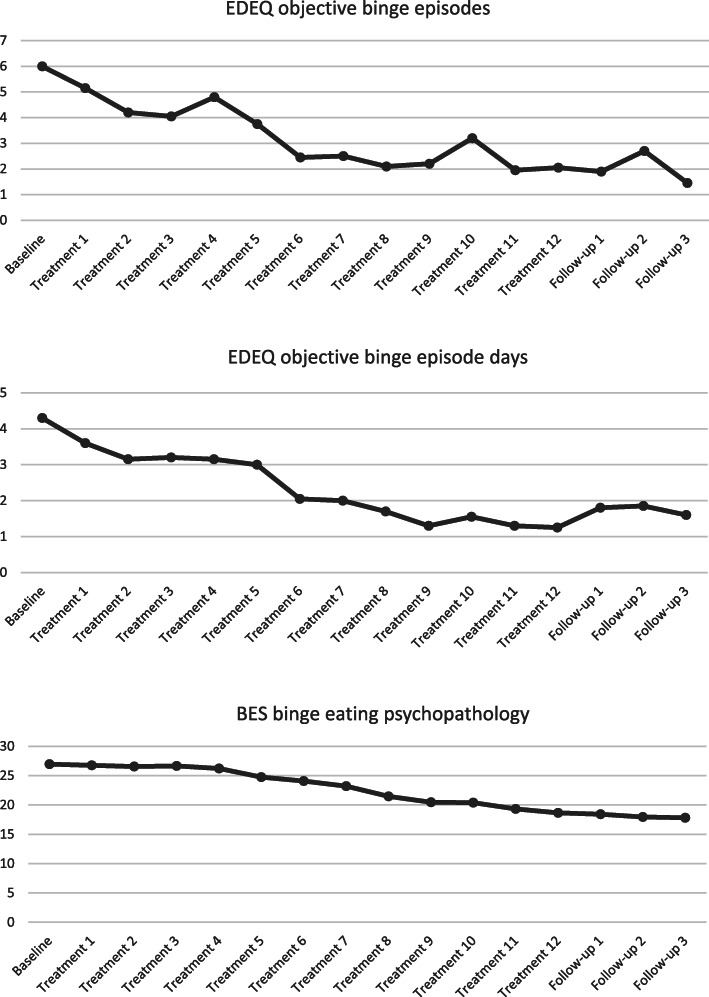
Mean EDEQ and BES pretherapy, therapy and follow up scores for the entire sample (*n* = 20)

Figure 2. and Table 5. outline combined EFT immediate intervention group and EFT wait-list control group within-group changes post the entire sample receiving treatment.

**Table 5:** Mean EDEQ and BES pretherapy, posttherapy and follow up scores for the entire sample (*n* = 20)

	Pretherapy^a.^ *M (SD)*	Posttherapy^b.^ *M (SD)*	*F*	*p*-value	η_p_^2^	Cohen’s *d*
Outcome						
EDEQ objective binge episodes	6.0 (4.92)	2.05 (2.39)	19.70	.001	.509	.99
EDEQ objective binge episode days	4.30 (2.23)	1.25 (1.33)	45.93	.001	.707	1.51
BES binge eating psychopathology	26.95 (8.30)	18.65 (10.45)	24.50	.001	.563	1.10
	Posttherapy^b.^ *M (SD)*	Follow-up^c.^ *M (SD)*	*F*	*p*-value	η_p_^2^	Cohen’s *d*
EDEQ objective binge episodes	2.05 (2.39)	1.45 (1.90)	1.21	.285	.060	.25
EDEQ objective binge episode days	1.25 (1.33)	1.60 (1.82)	.721	.406	.037	.19
BES binge eating psychopathology	18.65 (10.45)	17.80 (14.69)	.172	.683	.009	.09

*EDEQ* Eating Disorders Examination Questionnaire *BES* Binge Eating Scale; Week 0^a.^ Week 12^b.^ Week 24^c^

#### Objective binge episodes

There was a significant decrease in objective binge episode frequency scores measured over sixteen-time points including baseline (Week 0), treatment sessions (Week 1, 2, 3, 4, 5, 6, 7, 8, 9, 10, 11 and 12) (Weeks 1 to 12), and follow up at 1 month (Weeks 16), 2 months (Week 20) and 3 months (Week 24) [*F* 1, 2.33) = 4.43, *p* < .014, ηp^2^ = .189]. Mauchly’s Test of Sphericity indicated that the assumption of sphericity was violated [χ2(119) = 501.42, *p* < .001] and therefore, the Huynh-Feldt correction was used for the ANOVA. The analyses of the changes in objective binge episodes are shown in Table 5.

Pretherapy objective binge episodes significantly decreased from 6.00 (*SD* = 4.92) to 2.05 (*SD* = 2.39) posttherapy with a large effect size (*d* = .99). There was no significant difference between posttherapy and 3 months of follow-up scores, suggesting that treatment gains were maintained.

#### Objective binge episode days

There was a significant decrease in objective binge episode days scores measured over sixteen-time points including baseline (Week 0), treatment sessions (Week 1, 2, 3, 4, 5, 6, 7, 8, 9, 10, 11 and 12) (Weeks 1 to 12), and follow up at 1 month (Weeks 16), 2 months (Week 20) and 3 months (Week 24) [*F* 1, 8.44) = 8.78, *p* < .001, ηp^2^ = .316]. Mauchly’s Test of Sphericity indicated that the assumption of sphericity was violated [χ^2^(119) = 245.12, *p* < .001] and therefore, the Huynh-Feldt correction was used for the ANOVA. The analyses of the changes in objective binge episode days are shown in Table 5. Mean objective binge episode days decreased from 4.30 (*SD* = 2.23) pretherapy to 1.25 (*SD* = 1.33) posttherapy with a very large effect size (*d* = 1.51). There was no significant difference between posttherapy and 3 months of follow-up scores, suggesting that treatment gains were maintained.

#### Binge eating psychopathology

The BES demonstrated adequate internal consistency (α = .90). There was a significant decrease in binge eating psychopathology within-group scores measured over sixteen-time points including baseline (Week 0), treatment sessions (Week 1, 2, 3, 4, 5, 6, 7, 8, 9, 10, 11 and 12) (Weeks 1 to 12), and follow up at 1 month (Weeks 16), 2 months (Week 20) and 3 months (Week 24) [*F* 1, 4.04) = 9.84, *p* < .001, ηp^2^ = .341]. Mauchly’s Test of Sphericity indicated that the assumption of sphericity was violated [χ^2^(119) = 314.28, *p* < .001] and therefore, the Huynh-Feldt correction was used. Pretherapy mean BES scores significantly decreased from 26.95 (*SD* = 8.29) to 18.65 (*SD* = 10.45) posttherapy with a large effect size (*d* = 1.10) (see Table 5). Pre to posttherapy, the number of participants with severe binge eating range decreased from 14 to 6, and the number of non-binge eating participants increased from 3 to 11. There were 3 participants with mild to moderate binge eating at pre and posttherapy. At 3-month follow-up, there were 12 participants in the non-binge eating range, 1 mild to moderate and 7 in the severe range. There was no significant difference between posttherapy and 3-month follow-up scores, indicating that treatment gains were maintained.

## Discussion

To our knowledge, this is one of the first pilot randomised controlled trials to test the feasibility of individual EFT for BED. It builds upon more extensive previous research exploring the efficacy and feasibility of group therapy based EFT for BED [46] which used a non-randomised observational study, and a less extensive feasibility trial of individual therapy based EFT for BED which used a case study approach [47]. It was initially hypothesised that participants would be willing to be randomised; and an appropriate number of participants would be deemed eligible for the research. While a relatively small number of participants allocated to the EFT wait-list group expressed disappointment about not proceeding to treatment immediately, each expressed a strong willingness to continue involvement in the study. Further, while the recruitment phase was protracted due to a lower than expected take-up rate, this was mainly due to the relatively lower percentage of patients with binge eating disorder within the medical practices of referring general practitioner/primary care physicians. Future research could improve recruitment through liaison with relevant specialised eating disorder treatment and support services.

The second hypothesis was that that participation in EFT would result in higher participant treatment credibility and expectancy for improvement scores. Mean CEQ credibility scores remained high during early, mid and late therapy, and significant differences were not identified in scores from early to mid-therapy, mid to late therapy or early to late therapy. This finding suggests that EFT is a feasible treatment approach for BED. Our third hypothesis was that there would be a lower dropout rate using EFT for BED compared to more commonly used psychological treatment approaches. The 4.76% EFT for BED dropout rate in the current study compares favourably with dropout rates for other psychological treatment approaches for this condition including CBT - 11.1% [71] and 21.3% [72]; guided self-help cognitive behaviour therapy (CBTgsh) - 30% [73]; IPT - 8.6% [74] and 7% [75]; DBT - 4% [76]; and group psychodynamic interpersonal psychotherapy (GPIP) - 22.9% [72].

Our fourth hypothesis was that the immediate EFT treatment group participants would demonstrate a significantly greater degree of improvement in objective binge episodes, the number of days on which objective binge episodes occurred, and binge eating psychopathology, compared to participants in an EFT wait-list control group who had not received the treatment. Compared with the EFT wait-list control group, the immediate EFT intervention group showed significantly greater reductions in objective binge episodes with a large treatment effect (d = .98), objective binge days with a very large treatment effect (d = 1.39), and binge eating psychopathology with moderate treatment effect (d = .62). These findings provide support for the feasibility of EFT as a treatment approach for BED.

Our final and fifth hypothesis was that all participants receiving EFT (i.e. the EFT treatment group who initially received treatment, and also EFT wait-list group post receiving treatment), would experience significant improvements in objective binge episodes, the number of days on which objective binge episodes occurred, and binge eating psychopathology. There was a significant decrease from pretherapy to post-therapy in objective binge episodes with a large effect size (d = .99), objective binge episode days with a very large effect size (d = 1.51), and binge eating psychopathology with a large effect size (d = 1.10). The current findings compare favourably with posttherapy effect sizes for pooled primary outcome measures using bona fide vs non-bona fide therapies (d = .36) and CBT versus non-bona fide CBT (d = .30) for BED [77].

There was no significant difference between posttherapy and 3-month follow-up scores, indicating that objective binge episodes, objective binge episode days and binge eating psychopathology treatment gains were maintained. The number of participants classified as non-binge eating according to the BES in the current study was 11/20 (55%) at the end of treatment and 12/20 (60%) at 3 months follow up; and weekly mean objective binge episodes (in the previous 7 days) scores were 2.05 at the end of treatment and 1.45 at 3 months follow up. These findings compare favourably with results from a systematic review and network meta-analysis of the comparative effectiveness of treatments for BED [74] where abstinence rates for therapist-led, partially therapist-led and structured self-help variants of CBT ranged from 17.9 to 86.7% at the end of treatment, and 20.8 to 84.6% at twelve-month follow-up; and binge episodes (in the previous 28 days) ranged from an average of 11.9 to .04 at the end of treatment, and 16.2 and .5 at twelve-month follow up [73, 78, 79].

The following future sample size calculations were made using GPower 3.1.9.2 with alpha = .05, power = 0.95, 2 groups and 4-time points. Using our between-group effect size for objective binge eating frequency (*d* = .98), future studies comparing EFT to a wait-list control group will need a minimum sample size of 12. However, if comparing individual EFT with an active psychotherapy (*d* = 0.82) [79], future studies would require a minimum sample size of 320 participants to find an effect. Using our between-group effect size for days without bingeing and loss of control (d = 1.39), future studies comparing EFT to a wait-list control group will need a minimum sample size of 8 participants. If comparing EFT with an active psychotherapy (*d* = 1.04) [80], future studies will need a minimum of 70 participants to find an effect.

The main limitation of the current research is the relatively small sample size which may limit the extent to which the sample is representative of people with binge eating disorder. Additionally, the majority of the sample was female, and outcome measures were confined to self-report measures which limited a participant’s descriptions of attitudes and behaviours to those within their awareness. Posttreatment follow-up at 4, 8 and 12 weeks was also relatively short, which limited the analysis of participant trajectory and capacity to maintain gains. Finally, therapist effects cannot be ruled out given that the same therapist delivered the treatment; however, a recent investigation indicated that therapist effects account for only 5.8% of the variance in patient outcomes (e.g., [81]).

The present study has several implications. Firstly, it provides further preliminary evidence for the feasibility of EFT for BED and builds upon previous findings (e.g., [46, 47]). It also identified changes in objective binge episodes and days, and binge eating psychopathology that are theoretically important to EFT, including emotion and emotion regulation. The dropout rate was also relatively low compared to other psychological therapy interventions for BED, which indicates the acceptability of the EFT intervention.

## Conclusion

In conclusion, the evidence is emerging for the benefits of EFT for BED which has a focus on assisting clients in experiencing and processing unpleasant emotions and decreasing the reliance on an eating disorder as an emotional coping mechanism. Future research assessing EFT for BED needs to include a more extensive randomised control trial to assess efficacy with a larger sample size to establish causal conclusions and equal gender representation to improve the generalisability of findings. Consideration could also be given to a more extended follow-up period to improve the analysis of participant trajectory and capacity to maintain gains, and the use of more than one therapist to rule out therapist effects.

## Data Availability

The datasets used and/or analysed during the current study are available from the corresponding author on reasonable request.

## Abbreviations

ANOVA: Analyses of variance
BED: Binge-eating disorder
BES: Binge Eating Scale
BN: Bulimia nervosa
CBT: Cognitive behaviour therapy
CBTgsh: Guided self-help cognitive behaviour therapy
CEQ: Credibility Expectancy Questionnaire
CONSORT: Consolidated Standards of Reporting Trials
CT: Combined therapy
DBT: Dialectical behaviour therapy
DSM-5: Diagnostic and Statistical Manual of Mental Disorders: DSM-V
EDE-Q-6.0: Eating Disorder Examination Questionnaire 6.0
EFGT: Emotionally focused group therapy
EFT: Emotion-focused therapy
GPIP: Group psychodynamic interpersonal psychotherapy
IPT: Interpersonal therapy
NICE: National Institute of Clinical Excellence
QUT: Queensland University of Technology
RCT: Randomised control trial
SCID-5-RV: Structured Clinical Interview for DSM-5-Research Version
SCID-I: Structured Clinical Interview for DSM-IV Axis I Disorders
SD: Standard deviation
UHREC: University Human Research Ethics Committee

## References

[1] Kornstein S (2017). Epidemiology and recognition of binge-eating disorder in psychiatry and primary care. J Clin Psychiat.

[2] The National Eating Disorders Collaboration (2010). Eating disorders prevention, treatment and management: an evidence review.

[3] Hay P, Girosi F, Mond J (2015). Prevalence and sociodemographic correlates of DSM-5 eating disorders in the Australian population. J Eat Disord.

[4] American Psychiatric Association (2013). Diagnostic and statistical manual of mental disorders.

[5] Dingemans A, Danner U, Parks M (2017). Emotion regulation in binge eating disorder: a review. Nutri..

[6] Becker D, Jostmann N, Holland R (2018). Does approach bias modification really work in the eating domain? A commentary on Kakoschke et al. (2017). Add Behav.

[7] Blomquist KK, Grilo CM (2015). Family histories of anxiety in overweight men and women with binge eating disorder: a preliminary investigation. Compr Psychiatry.

[8] Araujo DMR, Santos GFDS, Nardi AE (2010). Binge eating disorder and depression: a systematic review. World J Biol Psychiatry.

[9] Udo T, McKee SA, Grilo CM (2014). Factor structure and clinical utility of the Beck depression inventory in patients with binge eating disorder and obesity. Gen Hosp Psychiat.

[10] American Psychiatric Association (2006). Treatment of patients with eating disorders. Third edition. Am J Psychiat.

[11] National Collaborating Centre for Mental Health. Eating disorders core interventions in the treatment and management of anorexia nervosa, bulimia nervosa, and related eating disorders. 2004. Place of publication not identified: British Psychological Society. 23346610

[12] Hilbert A, Petroff D, Herpertz S, Pietrowsky R, Tuschen-Caffier B, Vocks S (2019). Meta-analysis of the efficacy of psychological and medical treatments for binge-eating disorder. J Consult Clin Psychol.

[13] Linardon J (2018). Review of rates of abstinence following psychological or behavioural treatments for binge-eating disorder: meta-analysis. Int J Eat Disord.

[14] Gross J (1998). The emerging field of emotion regulation: an integrative review. Rev Gen Psychol.

[15] Bennett J, Greene G, Schwartz-Barcott D (2013). Perceptions of emotional eating behaviour. A qualitative study of college students. App..

[16] Bruch H. Eating disorders. Obesity, anorexia nervosa, and the person within: Routledge & Kegan Paul; 1974.

[17] Heatherton TF, Baumeister RF (1991). Binge eating as escape from self-awareness. Psychol Bull.

[18] Polivy J, Herman CP. Aetiology of binge eating: psychological mechanisms (1993). In C. G. Fairburn & G. T. Wilson (Eds.), binge eating: nature, assessment, and treatment (p. 173–205). Guilford Press.

[19] Pine CJ (1985). Anxiety and eating behaviour in obese and nonobese American Indians and white Americans. J Pers Soc Psychol.

[20] Leehr EJ, Krohmer K, Schag K, Dresler T, Zipfel S, Giel KE (2015). Emotion regulation model in binge eating disorder and obesity - a systematic review. Neurosci Biobehav Rev.

[21] Iacovino J, Gredysa D, Altman M, Wilfley D (2012). Psychological treatments for binge eating disorder. Curr Psychiatry Rep.

[22] Blocher-McCabe E, La Via M, Marcus MD, Thompson K (2004). Dialectical behaviour therapy for eating disorders. Handbook of eating disorders and obesity.

[23] Brockmeyer T, Skunde M, Wu M, Bresslein E, Rudofsky G, Herzog W, Friederich HC (2014). Difficulties in emotion regulation across the spectrum of eating disorders. Compr Psychiatry.

[24] Kenny T, Singleton C, Carter J (2017). Testing predictions of the emotion regulation model of binge-eating disorder. Int J Eat Disord..

[25] Svaldi J, Tuschen-Caffier B, Trentowska M, Caffier D, Naumann E (2014). Differential caloric intake in overweight females with and without binge eating: effects of a laboratory-based emotion-regulation training. Behav Res Ther.

[26] Peterson CB, Engel SG, Crosby RD, et al. Comparing integrative cognitive-affective therapy and guided self-help cognitive-behavioural therapy to treat binge-eating disorder using standard and naturalistic momentary outcome measures: a randomised controlled trial. Int J Eat Disord. 2020:1–10. 10.1002/eat.23324 PMC12931584 32583478

[27] Torres S, Sales C, Guerra M, Simões M, Pinto M, Vieira F (2020). Emotion-focused cognitive behavioural therapy in comorbid obesity with binge eating disorder: a pilot study of feasibility and long-term outcomes. Front Psychol.

[28] Dolhanty J, Greenberg LS (2007). Emotion-focused therapy in the treatment of eating disorders. Eur Psychother.

[29] Ivanova I, Watson J (2014). Emotion-focused therapy for eating disorders: enhancing emotional processing. Pers-Cent Exp Psychoth.

[30] Greenberg LS (2010). Emotion-focused therapy.

[31] Greenberg LS (2002). Emotion-focused therapy: coaching clients to work through their feelings.

[32] Elliott R, Watson JC, Goldman RN, Greenberg LS (2004). Learning emotion-focused therapy: the process experiential approach to change.

[33] Goldman R, Greenberg L, Angus L (2006). The effects of adding emotion-focused interventions to the client-centred relationship conditions in the treatment of depression. Psychother Res.

[34] Greenberg L, Watson J (2005). Emotion-focused therapy for depression.

[35] Robinson A, McCague E, Whissell C (2014). That chair work thing was great: a pilot study of group-based emotion-focused therapy for anxiety and depression. Pers-Cent Exp Psychot.

[36] Holowaty K, Paivio S (2012). Characteristics of client-identified helpful events in emotion-focused therapy for child abuse trauma. Psychother Res.

[37] Paivio S, Nieuwenhuis J (2001). Efficacy of emotion-focused therapy for adult survivors of child abuse: a preliminary study. J Trau Str.

[38] Paivio S, Pascual-Leone A (2010). Emotion-focused therapy for complex trauma.

[39] Shahar B, Bar-Kalifa E, Alon E (2017). Emotion-focused therapy for social anxiety disorder: results from a multiple-baseline study. J Con Clin Psychol.

[40] Timulak L, McElvaney J, Keogh D, Martin E, Clare P, Chepukova E (2017). Emotion-focused therapy for generalised anxiety disorder: an exploratory study. Psychot..

[41] Watson J, Greenberg L (2017). Emotion-focused therapy for generalised anxiety.

[42] Dolhanty J, Greenberg L (2009). Emotion-focused therapy in the treatment of eating disorders. Eur Psychot.

[43] Robinson AL, Dolhanty J, Greenberg L (2013). Emotion-focused family therapy for eating disorders in children and adolescents. Clin Psychol Psychother.

[44] Brennan MA, Emmerling ME, Whelton WJ (2015). Emotion-focused group therapy: addressing self-criticism in the treatment of eating disorders. Couns Psychother Res.

[45] Wnuk SM, Greenberg L, Dolhanty J (2015). Emotion-focused group therapy for women with symptoms of bulimia nervosa. Eat Disord.

[46] Compare A, Tasca GA (2016). The rate and shape of change in binge-eating episodes and weight: an effectiveness trial of emotionally focused group therapy for binge-eating disorder. Clin Psychol Psychother..

[47] Glisenti K, Strodl E, King R (2018). Emotion-focused therapy for binge-eating disorder: a review of six cases. Clini Psychol and Psychoth.

[48] Arain M, Campbell MJ, Cooper CL, Lancaster GA (2010). (2010). What is a pilot or feasibility study? A review of current practice and editorial policy. BMC Med Res Meth.

[49] Leon A, Davis L, Kraemer H (2011). The role and interpretation of pilot studies in clinical research. J Psychiatr Res.

[50] Fischer S, Peterson C (2015). Dialectical behaviour therapy for adolescent binge eating, purging, suicidal behaviour, and non-suicidal self-injury: a pilot study. Psychoth..

[51] Lewer M, Kosfelder J, Michalak J, Schroeder D, Nasrawi N, Vocks S (2017). Effects of a cognitive-behavioural exposure-based body image therapy for overweight females with binge eating disorder: a pilot study. J Eat Dis.

[52] Kelly A, Carter J (2014). Self-compassion training for binge eating disorder: a pilot randomised controlled trial. Psychol and Psychothy: Theo, Res and Prac.

[53] Efird J (2011). Blocked randomisation with randomly selected block sizes. Int J Environ Res Public Health.

[54] First MB, Williams JBW, Karg RS, Spitzer RL (2015). Structured clinical interview for DSM-5-research version (SCID-5 for DSM-5, research version; SCID-5-RV).

[55] Shankman S, Funkhouser C, Klein D, Davila J, Lerner D, Hee D (2018). Reliability and validity of severity dimensions of psychopathology assessed using the Structured Clinical Interview for DSM-5. Int J Meth Psychiatr Res.

[56] First M, Spitzer R, Gibbon M, Williams J (1996). Structured clinical interview for DSM-IV-TR Axis I disorders, research version, patient edition. (SCID-I/P).

[57] Ventura J, Liberman RP, Green MF, Shaner A, Mintz J (1998). Training and quality assurance with the structured clinical interview for DSM-IV (SCID-I/P). Psychiatry Res.

[58] Devilly G, Borkovec T (2000). Psychometric properties of the credibility/expectancy questionnaire. J Behav Ther Exp Psychiat.

[59] Fairburn CG, Beglin SJ (1994). Assessment of eating disorders: interview or self-report questionnaire?. Int J Eat Disord..

[60] Fairburn CG, Cooper Z, Fairburn CG, Wilson GT (1993). The eating disorder examination. Binge eating: nature, assessment and treatment.

[61] Rose JS, Vaewsorn A, Rosselli-Navarra F, Wilson G, Weissman R (2013). Test-retest reliability of the eating disorder examination-questionnaire (EDE-Q) in a college sample. J Eat Disord.

[62] Mond J, Hay P, Rodgers B, Owen C, Beumont P (2004). Validity of the eating disorder examination questionnaire (EDE-Q) in screening for eating disorders in community samples. Behav Res Ther.

[63] Reas DL, Grilo CM, Masheb RM (2006). Reliability of the eating disorder examination-questionnaire in patients with binge eating disorder. Behav Res Ther.

[64] Berg KC, Peterson CB, Frazier P, Crow SJ (2012). Psychometric evaluation of the eating disorder examination and eating disorder examination-questionnaire: a systematic review of the literature. Int J Eat Disord..

[65] Peterson C, Crosby R, Wonderlich S, Joiner T, Crow S, Mitchell J (2007). Psychometric properties of the eating disorder examination-questionnaire: factor structure and internal consistency. Int J Eat Disord..

[66] Gormally J, Black S, Daston S, Rardin D (1982). The assessment of binge eating severity among obese persons. Add Behav..

[67] Dezhkam M, Moloodi R, Mootabi F, Omidvar N (2009). Standardisation of the binge eating scale among an Iranian obese population. Iran J Psychiatry.

[68] Timmerman GM (1999). Binge eating scale: further assessment of validity and reliability. J Appl Biobehav Res.

[69] Carano A, De Berardis D, Gambi F, Di Paolo C, Campanella D, Pelusi L (2006). Alexithymia and body image in adult outpatients with binge eating disorder. Int J Eat Disord..

[70] Duarte C, Pinto-Gouveia J, Ferreira C (2015). Expanding binge eating assessment: validity and screening value of the binge eating scale in women from the general population. Eat Behav.

[71] Wilfley DE, Welch RR, Stein RI, Spurrell EB, Cohen LR, Saelens BE (2002). A randomised comparison of group cognitive-behavioural therapy and group interpersonal psychotherapy for the treatment of overweight individuals with binge-eating disorder. Arch Gen Psychiatry.

[72] Tasca GA, Ritchie K, Conrad G, Balfour L, Gayton J, Lybanon V, Bissada H (2006). Attachment scales predict outcome in a randomised controlled trial of two group therapies for binge eating disorder: an aptitude by treatment interaction. Psychother Res.

[73] Peterson C, Mitchell J, Engbloom S, Nugent S, Mussell M, Miller J (1998). Group cognitive-behavioural treatment of binge eating disorder: a comparison of therapist-led versus self-help formats. Int J Eat Disord..

[74] Peat C, Berkman N, Lohr K, Brownley K, Bann C, Cullen K (2017). Review of comparative effectiveness of treatments for binge-eating disorder: systematic review and network meta-analysis. Eur Eat Disord Rev.

[75] Wilson GT, Wilfley DE, Agras WS, Bryson SW (2010). Psychological treatments of binge eating disorder. Arch Gen Psychiatry.

[76] Safer DL, Robinson AH, Jo B (2010). Outcomes from a randomised controlled trial of group therapy for binge eating disorder: comparing dialectical behaviour therapy adapted for binge eating to an active comparison group therapy. Behav Ther.

[77] Spielmans G, Benish S, Marin C, Bowman W, Menster M, Wheeler A (2013). Specificity of psychological treatments for bulimia nervosa and binge eating disorder? A meta-analysis of direct comparisons. Clin Psychol Rev.

[78] Peterson C, Mitchell J, Engbloom S, Nugent S, Mussell M, Crow S, Thuras P (2001). Self-help versus therapist-led group cognitive-behavioural treatment of binge eating disorder at follow-up. Int J Eat Disord..

[79] Peterson C, Mitchell J, Crow S, Crosby R, Wonderlich S (2009). The efficacy of self-help group treatment and therapist-led group treatment for binge eating disorder. Am J Psychiatry.

[80] Vocks S, Tuschen-Caffier B, Pietrowsky R, Rustenbach SJ, Kersting A, Herpertz S (2010). Meta-analysis of the effectiveness of psychological and pharmacological treatments for binge eating disorder. Int J Eat Disord..

[81] Saxon D, Firth N, Barkham M (2017). The relationship between therapist effects and therapy delivery factors: therapy modality, dosage, and non-completion. Admin Pol Ment Health.

